# Factors influencing intraoperative and postoperative complication occurrence: A series of 1135 periodontal and implant‐related surgeries

**DOI:** 10.1002/cre2.849

**Published:** 2024-02-06

**Authors:** Gary M. Blyleven, Thomas M. Johnson, Kimberly Ann Inouye, Brian W. Stancoven, Adam R. Lincicum

**Affiliations:** ^1^ Department of Periodontics Army Postgraduate Dental School, Postgraduate Dental College, Uniformed Services University of the Health Sciences, Fort Eisenhower Augusta Georgia USA

**Keywords:** alveolar ridge augmentation, intraoperative complications, postoperative complications, treatment outcome

## Abstract

**Objectives:**

In periodontology, it is widely recognized that evidence characterizing the incidence and effect of treatment complications is lacking. The objective of this study was to assess the influence of operator‐, procedure‐, patient‐, and site‐associated factors on intraoperative and postoperative complication occurrence.

**Material and Methods:**

A single investigator reviewed records of patients treated by eight periodontics residents from July 2018 through June 2022. For each procedure, the investigator recorded each intraoperative and postoperative complication or indicated that no complication had occurred. These outcomes were analyzed against a panel of explanatory covariates. In addition, the severity of each postoperative complication was assessed using a standardized grading system.

**Results:**

A total of 1135 procedures were included in the analysis. Intraoperative and postoperative complications were identified in 2.8% and 15.2% of procedures, respectively. The most common intraoperative complications were Schneiderian membrane perforation (1.3%) and gingival flap perforation/tear (1%), and the most common postoperative complications were dentin hypersensitivity (2.6%), excessive pain (2.5%), and infection (2.2%). Subepithelial connective tissue graft (odds ratio [OR]: 3.2, 95% confidence interval [CI]: 1.6, 6.1; *p* < .001), guided bone regeneration (OR: 3.0, 95% CI: 1.4, 6.5; *p* = .004), and guided bone regeneration with implant placement (OR: 3.1, 95% CI: 1.3, 7.6; *p* = .011) were associated with higher odds of postoperative complication, whereas lateral sinus elevation (OR: 102.5, 95% CI: 12.3, 852.9; *p* < .001), transalveolar sinus elevation (OR: 22.4, 95% CI: 2.2, 224.5; *p* = .008), open flap debridement (OR: 36.4, 95% CI: 3.0, 440.7; *p* = .005), and surgically facilitated orthodontic therapy (OR: 20.5, 95% CI: 1.2, 358.4; *p* = .039) were associated with higher odds of intraoperative complication occurrence.

**Conclusions:**

Consistent with previous reports, procedure type appears to be the predominant factor driving complication occurrence. As analyses of treatment complications increase, individualized risk‐benefit assessments will become progressively meaningful for patients.

**Abbreviations**
ADM: acellular dermal matrix
BRG: bone replacement graft
CL: crown lengthening
EPG: epithelialized palatal graft
GBR: guided bone regeneration
IIP: immediate implant placement
LPT: laser periodontal therapy
OFD: open flap debridement
SES: sinus elevation surgery
SFOT: surgically facilitated orthodontic therapy

## 1 INTRODUCTION

According to the American Dental Education Association, a principal motivation dentists report for choosing the dental profession is a desire to transform the lives of patients by restoring oral health (American Dental Education Association, 2023). Indeed, reducing suffering and restoring health are among the most rewarding aspects of clinical dentistry. Intuitively, virtually all dentists seek to protect patients from adverse treatment outcomes and strive to achieve excellent therapeutic results. The foundation of this enduring ideal arose in antiquity and remains preserved in the Latin axiom *primum non nocere*—first do no harm (Gillon, 1985). Nevertheless, practicing clinicians understand the tension between beneficence (doing or producing good) and non‐maleficence (doing no harm). Nearly every clinical protocol in medicine and dentistry is subject to error, complication occurrence, and adverse effects (Institute of Medicine US Committee on Quality of Health Care in America, 2000; Zucchelli et al., 2023). To deliver healthcare is to open the possibility of an untoward outcome, and clinicians in practice must be equipped to avoid, recognize, manage, and mitigate the impact of treatment complications and adverse effects.

Various factors have been shown to influence healing, postoperative morbidity, and complication occurrence following dentoalveolar, periodontal, and dental implant surgeries. These include cigarette smoking (Askar et al., 2019; Schwartz‐Arad et al., 2017), diabetes (Askar et al., 2019), gender (Eli et al., 2000; Schwartz‐Arad et al., 2017), age (Askar et al., 2019; Chuang et al., 2007), oral contraceptive use (Xu et al., 2015), procedure type (Askar et al., 2019; Canakçi & Canakçi, 2007; López et al., 2011; Mei et al., 2016), operator experience (López et al., 2011; Mei et al., 2016), surgical complexity (Bui et al., 2003), extent and duration of surgery (López et al., 2011; Tan et al., 2014), perioperative antibiotic use (Askar et al., 2019; Esposito et al., 2013; Lodi et al., 2021), and psychological factors such as stress and anxiety (George et al., 1980; Wang et al., 2017). The purpose of this retrospective observational study was to assess the influence of operator‐, procedure‐, patient‐, and site‐associated factors on the incidence of intraoperative and postoperative complications related to periodontal and implant surgery.

## 2 MATERIALS AND METHODS

The Dwight David Eisenhower Army Medical Center Human Research Protections Office reviewed and approved this protocol (#21‐14188/945052) in accordance with the exemption criteria described in 32CFR§219.104(d), Category 4. Data were collected in such a manner that the identities of human subjects were not readily discernible directly or through identifiers linked with subjects. The procedures evaluated in this study were performed by eight residents in the Department of Periodontics, Army Postgraduate Dental School, Postgraduate Dental College, Fort Eisenhower, Georgia, between July 1, 2018, and June 10, 2022. Before treatment, each patient completed an informed consent process involving verbal and written components. This investigation complies with the Declaration of Helsinki and the Strengthening the Reporting of Observational Studies in Epidemiology (STROBE) guidelines.

### 2.1 Inclusion criteria

To be included in this study, a patient needed to have received delayed implant placement, immediate implant placement (IIP), implant plus autogenous soft tissue graft, implant plus acellular dermal matrix (ADM), implant plus guided bone regeneration (GBR), GBR alone, transalveolar sinus elevation surgery (SES) ± implant placement, lateral SES ± implant placement, epithelialized palatal graft (EPG), subepithelial connective tissue graft (SCTG), acellular dermal matrix (ADM), guided tissue regeneration (GTR) ± bone replacement graft (BRG), BRG alone, osseous surgery, open flap debridement (OFD), crown lengthening (CL), gingivectomy/gingivoplasty, distal wedge, surgically facilitated orthodontic therapy (SFOT), or laser periodontal therapy (LPT). In addition, the patient needed to have been followed in the Department of Periodontics for at least 6 weeks.

### 2.2 Exclusion criteria

Patients who received a surgical procedure other than the defined procedure types, patients with ambiguous or incomplete records, and patients with less than 6 weeks follow‐up were excluded from this analysis.

The primary outcomes of interest consisted of intraoperative and postoperative complication occurrence. These outcome variables were compared against a panel of operator‐, procedure‐, patient‐, and site‐related covariates. Specifically, independent variables included operator, year of training, patient age, patient gender, diabetic status, current smoking status, region (anterior or posterior), site type (single tooth position or multiple adjacent tooth positions), peri‐operative antibiotic use, and procedure type (defined in the inclusion criteria). In addition to recording occurrence or absence of a postoperative complication, the investigator scored postoperative complications according to the grading scale of Askar and colleagues (2019). Smoking status and diabetic status each had two levels—current smoker versus nonsmoker and diabetic versus nondiabetic. A diagnosis of diabetes was confirmed in the patient record. Patients who smoked at least one cigarette per day were considered smokers.

### 2.3 Intraoperative and postoperative complication types

Intraoperative complication types defined in this study included mucoperiosteal flap perforation/tear, Schneiderian membrane perforation, retained root fragment, excessive intraoperative bleeding, thermal injury, iatrogenic damage to adjacent structures, and the need to abort surgery for other reasons. Postoperative complication types defined in this study included dentin hypersensitivity, excessive postoperative swelling, postoperative bleeding, wound dehiscence, membrane exposure, postoperative infection, graft necrosis, excessive postoperative pain, ecchymosis, altered neurosensory function, sinusitis, trismus, urticaria, gastrointestinal disturbance, and early implant failure (osseointegration failure).

Postoperative pain was considered excessive if the provider indicated in the patient record that the pain exceeded the expected level, extended the patient's convalescence period due to discomfort, or prescribed additional analgesics. Postoperative swelling was considered a complication if the provider indicated in the patient record that swelling exceeded the expected level, persisted for at least 2 weeks, or required additional appointments for monitoring in the early postoperative period. Postoperative bleeding was considered a complication if the patient returned to the clinic with bleeding requiring an intervention to achieve hemostasis or if the patient reported bleeding to the surgeon telephonically but was able to achieve hemostasis with gauze and pressure without returning to the clinic. Implant failure within 6 months of placement was considered a postoperative complication for the purpose of this analysis.

### 2.4 Statistical analyses

Descriptive and inferential statistics were calculated for all variables using IBM SPSS Statistics for Windows v.28, and statistical significance was assessed at an alpha level of 0.05. Binomial logistic regression analyses were used to ascertain the effects of operator‐, procedure‐, patient‐, and site‐related explanatory variables (listed above) on intraoperative and postoperative complication occurrence. For each regression model, omnibus tests were used to assess model fit. Linearity of the continuous variable (age) with respect to the logit of the dependent variables was assessed via the Box‐Tidwell procedure. Odds ratios (ORs) and 95% confidence intervals (CIs) were determined. Use of binomial logistic regression required definition of reference categories for each categorical independent variable—procedure type, antibiotic, and operator. Delayed implant placement, a relatively simple and low‐risk procedure, was selected as the reference category for procedure type. “No antibiotic” served as a non‐intervention reference category for antibiotics, and Operator 1 served as the reference category for the operator variable.

## 3 RESULTS

Table 1 summarizes the study sample and presents descriptive statistics for the dependent and independent variables, and Table 2 presents the frequencies of intraoperative and postoperative complications for each procedure type. In this study, male patients outnumbered female patients by a ratio of nearly 4:1. Less than 10% of the patients were smokers, and only 3.5% of patients were diabetic. In 680 (59.9%) procedures, the patient received no perioperative antibiotics. Second‐year and third‐year residents performed 507 (44.7%) and 628 (55.3%) of the procedures, respectively.

**Table 1:** Dependent and independent variable frequencies in 1135 evaluated procedures.

Variable	*n*	%	Variable	*n*	%
**Operator**			**Dental arch**		
Operator 1	185	16.3	Maxillary arch	565	49.8
Operator 2	128	11.3	Mandibular arch	570	50.2
Operator 3	127	11.2	**Region**		
Operator 4	117	10.3	Posterior	882	77.7
Operator 5	160	14.1	Anterior	253	22.3
Operator 6	127	11.2	**Procedure type**		
Operator 7	150	13.2	Crown lengthening	181	15.9
Operator 8	141	12.4	Delayed implant	165	14.5
**Year of training**			ARP	155	13.7
Second year	507	44.7	SCTG	103	9.1
Third year	628	55.3	LPT	68	6.0
**Patient gender**			GBR	60	5.3
Female	290	25.6	GTR ± BRG	55	4.8
Male	845	74.4	ADM	43	3.8
**Smoking status**			Immediate implant	42	3.7
Nonsmoker	1028	90.6	Osseous surgery	41	3.6
Current smoker	107	9.4	Implant + GBR	38	3.3
**Diabetic status**			EPG	29	2.6
Nondiabetic	1095	96.5	Distal wedge	28	2.5
Diabetic	40	3.5	Lateral SES ± implant	26	2.3
**Perioperative antibiotic use**			Transalveolar SES ± implant	25	2.2
No antibiotic	680	59.9	GV/gingivoplasty	22	1.9
Amoxicillin	313	27.6	BRG alone	17	1.5
Amoxicillin + clavulanate	89	7.8	OFD	11	1.0
Clindamycin	31	2.7	SFOT	9	0.8
Doxycycline	16	1.4	Implant + EPG or SCTG	9	0.8
Azithromycin	5	0.4	Implant + ADM	8	0.7
Amoxicillin + metronidazole	1	0.1	**Postoperative complication occurrence**		
**Intraoperative complication occurrence**			No complication	962	84.8
No complication	1104	97.2	Dentin hypersensitivity	29	2.6
Schneiderian membrane perforation	15	1.3	Excessive postoperative pain	28	2.5
Flap perforation or tear	11	1.0	Postoperative infection	25	2.2
Excessive bleeding	1	0.09	Wound dehiscence	18	1.6
Retained root fragment	1	0.09	Membrane exposure	18	1.6
Thermal injury	1	0.09	Osseointegration failure	12	1.1
Tachycardia	1	0.09	Delayed wound healing	16	1.4
Unfavorable implant osteotomy position	1	0.09	Postoperative swelling	9	0.8
**Postoperative complication grade**			Altered sensation, temporary	6	0.5
No complication	962	84.8	Graft necrosis/failure to vascularize	6	0.5
Localized complication, no adverse effect on success	55	4.8	Postoperative bleeding	1	0.1
Localized complication, adverse effect on success	79	7.0	Ecchymosis	3	0.3
Localized/systemic complication, impaired daily activities	39	3.4	Trismus	2	0.2

Abbreviations: ADM, acellular dermal matrix; ARP, alveolar ridge preservation; BRG, bone replacement graft; EPG, epithelialized palatal graft; GBR, guided bone regeneration; GTR, guided tissue regeneration; GV, gingivectomy; LPT, laser periodontal therapy; OFD, open flap debridement; SCTG, subepithelial connective tissue graft; SES, sinus elevation surgery; SFOT, surgically facilitated orthodontic treatment.

**Table 2:** Complication occurrence by procedure type.

Procedure	*n*	%	Procedure	*n*	%
**Crown lengthening (*n*** = **181)**			**Immediate implant placement (*n*** = **42)**		
Intraoperative complications			Postoperative complications		
No complication	178	98.3	No complication	34	81.0
Flap perforation or tear	2	1.1	Early implant failure	3	7.1
Excessive bleeding	1	0.6	Postoperative infection	3	7.1
Postoperative complications			Excessive postoperative swelling	1	2.4
No complication	157	86.7	Delayed wound healing	1	2.4
Dentin hypersensitivity	11	6.1	**Osseous surgery (*n*** = **41)**		
Excessive postoperative pain	4	2.2	Postoperative complications		
Postoperative infection	3	1.7	No complication	35	85.4
Wound dehiscence	2	1.1	Dentin hypersensitivity	3	7.3
Delayed wound healing	2	1.1	Excessive postoperative pain	2	4.9
Transient paresthesia/altered sensation	1	0.6	Wound dehiscence	1	2.4
Trismus	1	0.6	**Implant + guided bone regeneration (*n*** = **38)**		
**Delayed implant placement (*n*** = **165)**			Intraoperative complications		
Intraoperative complications			No complication	36	94.7
No complication	163	98.8	Flap perforation or tear	2	5.3
Postoperative bleeding	2	1.2	Postoperative complications		
Postoperative complications			No complication	26	68.4
No complication	147	89.1	Membrane exposure	3	7.9
Early implant failure	8	4.8	Postoperative infection	2	5.3
Postoperative infection	4	2.4	Excessive postoperative pain	2	5.3
Wound dehiscence	3	1.8	Excessive postoperative swelling	2	5.3
Excessive postoperative pain	2	1.2	Delayed wound healing	2	5.3
Delayed wound healing	1	0.6	Wound dehiscence	1	2.6
**Alveolar ridge preservation (*n*** = **155)**			**Epithelialized palatal graft (*n*** = **29)**		
Intraoperative complications			Postoperative complications		
No complication	152	98.1	No complication	26	89.7
Schneiderian membrane perforation	2	1.3	Delayed wound healing	2	6.9
Other	1	0.6	Excessive postoperative pain	1	3.4
Postoperative complications			**Distal wedge (*n*** = **28)**		
No complication	142	91.6	Postoperative complications		
Excessive postoperative pain	4	2.6	No complication	25	89.3
Delayed wound healing	3	1.9	Wound dehiscence	2	7.1
Postoperative infection	2	1.3	Trismus	1	3.6
Excessive postoperative swelling	1	0.6	**Lateral sinus elevation surgery ± implant (*n*** = **26)**		
Excessive postoperative bleeding	1	0.6	Intraoperative complications		
Wound dehiscence	1	0.6	No complication	16	61.5
Ecchymosis	1	0.6	Schneiderian membrane perforation	10	38.5
**Subepithelial connective tissue graft (*n*** = **103)**			Postoperative complications		
Intraoperative complications			No complication	24	92.3
No complication	98	95.1	Wound dehiscence	2	7.7
Flap perforation or tear	5	4.9			
**Transalveolar sinus elevation surgery ± implant (*n*** = **25)**					
Postoperative complications			Intraoperative complications		
No complication	76	73.8	No complication	22	88.0
Excessive postoperative pain	6	5.8	Schneiderian membrane perforation	3	12.0
Dentin hypersensitivity	4	3.9	Postoperative complications		
Excessive postoperative swelling	4	3.9	No complication	22	88.0
Postoperative infection	4	3.9	Early implant failure	1	4.0
Graft necrosis	4	3.9	Postoperative infection	1	4.0
Delayed wound healing	2	1.9	Wound dehiscence	1	4.0
Ecchymosis	2	1.9	**Bone replacement graft (*n*** = **17)**		
Wound dehiscence	1	1.0	Intraoperative complications		
**Laser periodontal therapy (*n*** = **68)**			No complication	16	94.1
Postoperative complications			Flap perforation or tear	1	5.9
No complication	61	89.7	Postoperative complications		
Dentin hypersensitivity	6	8.8	No complication	15	88.2
Excessive postoperative pain	1	1.5	Dentin hypersensitivity	1	5.9
**Guided bone regeneration (*n*** = **60)**			Wound dehiscence	1	5.9
Postoperative complications			**Open flap debridement (*n*** = **11)**		
No complication	44	73.3	Intraoperative complications		
Excessive postoperative pain	4	6.7	No complication	9	81.8
Postoperative infection	3	5.0	Flap perforation or tear	1	9.1
Transient paresthesia/altered sensation	3	5.0	Anxiety‐related tachycardia	1	9.1
Wound dehiscence	2	3.3	Postoperative complications		
Membrane exposure	2	3.3	No complication	10	90.9
Postoperative swelling	1	1.7	Dentin hypersensitivity	1	9.1
Delayed wound healing	1	1.7	**Surgically facilitated orthodontic therapy (*n*** = **9)**		
**Guided tissue regeneration ± bone replacement graft (*n*** = **55)**			Intraoperative complications		
Postoperative complications			No complication	8	88.9
No complication	47	85.5	Burn injury from piezosurgery instrument	1	11.1
Membrane exposure	3	5.5	Postoperative complications		
Dentin hypersensitivity	2	3.6	No complication	7	77.8
Delayed wound healing	2	3.6	Transient paresthesia/altered sensation	1	11.1
Postoperative infection	1	1.8	Wound dehiscence	1	11.1
**Acellular dermal matrix (*n*** = **43)**			**Implant + acellular dermal matrix (*n*** = **8)**		
Postoperative complications			Postoperative complications		
No complication	36	83.7	No complication	6	75.0
Postoperative infection	2	4.7	Excessive postoperative swelling	1	12.5
Graft necrosis/failure to vascularize	2	4.7	Excessive postoperative pain	1	12.5
Dentin hypersensitivity	1	2.3	**Implant + autologous soft tissue graft (*n*** = **9)**		
Delayed wound healing	1	2.3	No intraoperative or postoperative complication	9	100
Excessive postoperative pain	1	2.3	**Gingivectomy/gingivoplasty (*n*** = **22)**		
			No intraoperative or postoperative complication	22	100

Of 1135 evaluated procedures, 31 (2.8%) resulted in an intraoperative complication and 173 (15.2%) resulted in a postoperative complication. Of the 173 postoperative complications, 55 (31.8%) were localized complications accompanied by no adverse effects on the success of the procedure (grade 1), 79 (45.7%) were localized complications that did adversely influence the success of the procedure (grade 2), and 39 (22.5%) were localized or systemic complications that temporarily impaired the patient's daily routine (grade 3). No complication in this sample required hospitalization of the patient (grade 4) or resulted in irreversible damage to an anatomic structure (grade 5).

**Figure 1:**
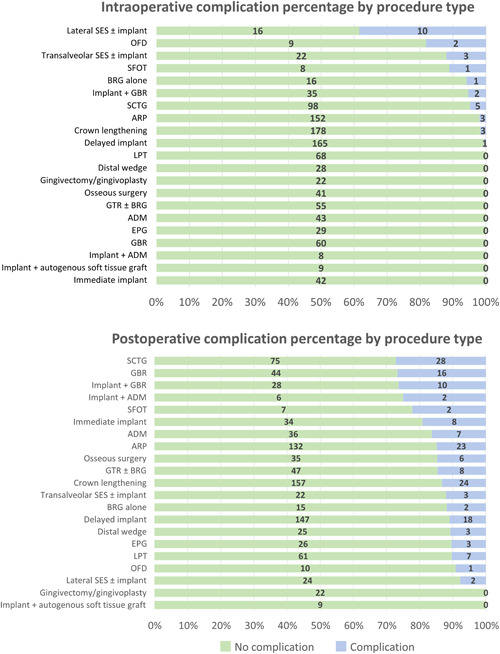
Intraoperative and postoperative complication percentages by procedure type. In binomial logistic regression analyses, lateral SES, transalveolar SES, and open flap debridement were associated with significantly higher odds of intraoperative complication occurrence. Subepithelial connective tissue graft, guided bone regeneration, and implant plus guided bone regeneration were associated with significantly higher odds of postoperative complication occurrence. The number in each bar indicates the actual number of events observed. ADM, acellular dermal matrix; ARP, alveolar ridge preservation; BRG, bone replacement graft; EPG, epithelialized palatal graft; GBR, guided bone regeneration; GTR, guided tissue regeneration; GV, gingivectomy, LPT, laser periodontal therapy; OFD, open flap debridement; SCTG, subepithelial connective tissue graft; SES, sinus elevation surgery; SFOT, surgically facilitated orthodontic therapy.

The most common postoperative complications (Figure 1) observed in this study were dentin hypersensitivity (29, 2.6%), excessive pain (28, 2.5%), and infection (25, 2.2%). Of the 29 occurrences of dentin hypersensitivity, 11 (37.9%) were associated with CL, six (20.7%) were associated with LPT, and four (13.8%) were associated with SCTG. The remaining eight occurrences of dentin hypersensitivity were noted following osseous surgery, OFD, BRG, GTR, and ADM. Of the 28 excessive pain episodes, six (21.4%) were recorded after SCTG, four (14.3%) after GBR, four (14.3%) after CL, and four (14.3%) after EPG procedures. The remaining 10 occurrences of excessive pain were associated with implant placement + GBR or ADM, ADM alone, or osseous surgery. Of the 25 postoperative infections, four (16.0%) occurred after SCTG, four (16.0%) occurred after delayed implant placement, and three (12.0%) occurred after IIP. Implant placement + GBR, GBR alone, transalveolar SES, ARP, ADM, and CL accounted for the remaining postoperative infections.

The most common intraoperative complications (Figure 1) observed in this study were Schneiderian membrane perforation (15, 1.3%) and mucoperiosteal flap perforation/tear (11, 1.0%). Ten (66.7%) Schneiderian membrane perforations occurred during lateral SES, three (20.0%) during transalveolar SES, and two (13.3%) during tooth extraction with ARP. Five (45.4%) mucoperiosteal flap perforations/tears occurred during SCTG procedures, two (18.2%) during implant placement with simultaneous GBR, and two (18.2%) during transalveolar SES.

**Figure 2:**
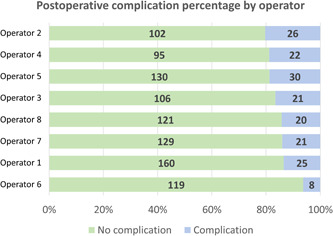
Postoperative complication percentage by operator. In a binomial logistic regression model including operator as the only variable, Operator 6 was associated with significantly lower odds of postoperative complication occurrence. Thus, operator was included as an independent variable in the final model. However, in the final model, this effect was not statistically significant. The number in each bar indicates the actual number of events observed.

A binomial logistic regression analysis was completed to determine whether operator‐, patient‐, site‐, and procedure‐related factors predicted postoperative complication occurrence. The model was built in a hierarchical fashion. Omnibus tests of model coefficients were used to eliminate from the model factors that were not significant predictors of the outcome. Only operator (Figure 2) and procedure type remained in the final model, and in the model including both of these variables, only procedure type was a statistically significant predictor of postoperative complication occurrence (Table 3). The interaction term between clinician and procedure type was not statistically significant. The final logistic regression model was statistically significant, *χ* ^2^(27) = 52.8, *p* = .002, explaining 8% (Nagelkerke R^2^) of the variance in postoperative complication occurrence. SCTG (OR: 3.2, 95% CI: 1.6, 6.1; *p* < .001), GBR (OR: 3.0, 95% CI: 1.4, 6.5; *p* = .004), and GBR with implant placement (OR: 3.1, 95% CI: 1.3, 7.6; *p* = .011) were associated with higher odds of postoperative complication.

**Table 3:** Binomial logistic regression predicting postoperative complication occurrence.

							95% CI for Exp(B)	
	*B*	*SE*	Wald	*df*	Sig.	Exp(B)	Lower	Upper
Operator			13.295	7	0.065			
Operator 2	0.508	0.319	2.540	1	0.111	1.662	0.890	3.103
Operator 3	0.271	0.330	0.676	1	0.411	1.311	0.687	2.503
Operator 4	0.502	0.330	2.313	1	0.128	1.652	0.865	3.156
Operator 5	0.412	0.302	1.858	1	0.173	1.510	0.835	2.732
Operator 6	−0.815	0.430	3.585	1	0.058	0.443	0.190	1.029
Operator 7	0.030	0.326	0.009	1	0.926	1.031	0.545	1.951
Operator 8	0.133	0.332	0.160	1	0.689	1.142	0.595	2.190
Procedure type			28.606	20	0.096			
Immediate implant	0.611	0.469	1.697	1	0.193	1.842	0.735	4.617
Implant + GBR	1.140	0.451	6.392	1	0.011	3.125	1.292	7.560
Implant + EPG or SCTG	−19.073	13354.423	0.000	1	0.999	0.000	0.000	–
Implant + ADM	1.062	0.865	1.506	1	0.220	2.892	0.530	15.766
Transalveolar SES ± implant	0.165	0.669	0.061	1	0.805	1.179	0.318	4.376
Lateral SES ± implant	−0.398	0.782	0.259	1	0.611	0.672	0.145	3.108
ARP	0.362	0.339	1.136	1	0.286	1.436	0.738	2.791
GBR	1.109	0.388	8.163	1	0.004	3.032	1.417	6.491
EPG	−0.038	0.664	0.003	1	0.954	0.962	0.262	3.538
SCTG	1.153	0.338	11.642	1	<0.001	3.168	1.633	6.144
ADM	0.399	0.487	0.671	1	0.413	1.490	0.574	3.868
BRG alone	−0.071	0.797	0.008	1	0.929	0.931	0.195	4.445
GTR ± BRG	0.335	0.461	0.529	1	0.467	1.398	0.567	3.447
Osseous surgery	0.307	0.514	0.357	1	0.550	1.359	0.497	3.719
OFD	−0.107	1.087	0.010	1	0.922	0.898	0.107	7.564
Crown lengthening	0.186	0.335	0.308	1	0.579	1.204	0.625	2.322
GV/gingivoplasty	−18.916	8438.745	0.000	1	0.998	0.000	0.000	–
Distal wedge	−0.122	0.666	0.034	1	0.854	0.885	0.240	3.263
SFOT	0.856	0.848	1.020	1	0.312	2.355	0.447	12.405
LPT	0.048	0.475	0.010	1	0.920	1.049	0.414	2.661
Constant	−2.279	0.320	50.655	1	<0.001	0.102		

*Note*: Reference categories were Operator 1 and delayed implant placement.

Abbreviations: ADM, acellular dermal matrix; ARP, alveolar ridge preservation; BRG, bone replacement graft; CI, confidence interval; EPG, epithelialized palatal graft; GBR, guided bone regeneration; GTR, guided tissue regeneration; GV, gingivectomy, LPT, laser periodontal therapy; OFD, open flap debridement; SCTG, subepithelial connective tissue graft; SES, sinus elevation surgery; SFOT, surgically facilitated orthodontic therapy.

A binomial logistic regression analysis was completed to determine whether operator‐, patient‐, site‐, and procedure‐related factors predicted intraoperative complication occurrence. The model was built in a hierarchical fashion. Omnibus tests of model coefficients were used to eliminate from the model factors that were not significant predictors of the outcome. Only procedure type was found to be a statistically significant predictor of intraoperative complication occurrence (Table 4). The final logistic regression model was statistically significant, *χ* ^2^(20) = 79.0, *p* < .001, explaining 30.3% (Nagelkerke R^2^) of the variance in intraoperative complication occurrence. Lateral SES (OR: 102.5, 95% CI: 12.3, 852.9; *p* < .001), transalveolar SES (OR: 22.4, 95% CI: 2.2, 224.5; *p* = .008), OFD (OR: 36.4, 95% CI: 3.0, 440.7; *p* = .005), and SFOT (OR: 20.5, 95% CI: 1.2, 358.4; *p* = .039) were associated with higher odds of intraoperative complication occurrence.

**Table 4:** Binomial logistic regression predicting intraoperative complication occurrence.

							95% CI for EXP(B)	
	*B*	*SE*	Wald	*df*	Sig.	Exp(B)	Lower	Upper
Procedure type			49.294	20	<0.001			
Immediate implant	−16.103	6201.910	0.000	1	0.998	0.000	0.000	–
Implant + GBR	2.209	1.238	3.183	1	0.074	9.111	0.804	103.224
Implant + autogenous soft tissue graft	−16.103	13397.657	0.000	1	0.999	0.000	0.000	–
Implant + ADM	−16.103	14210.361	0.000	1	0.999	0.000	0.000	–
Transalveolar SES ± implant	3.107	1.177	6.973	1	0.008	22.364	2.228	224.515
Lateral SES ± implant	4.630	1.081	18.343	1	<0.001	102.500	12.319	852.878
ARP	1.175	1.160	1.025	1	0.311	3.237	0.333	31.453
GBR	−16.103	5188.890	0.000	1	0.998	0.000	0.000	–
EPG	−16.103	7463.647	0.000	1	0.998	0.000	0.000	–
SCTG	2.124	1.103	3.710	1	0.054	8.367	0.963	72.668
ADM	−16.103	6129.371	0.000	1	0.998	0.000	0.000	–
BRG alone	2.327	1.438	2.618	1	0.106	10.250	0.612	171.781
GTR ± BRG	−16.103	5419.619	0.000	1	0.998	0.000	0.000	–
Osseous surgery	−16.103	6277.087	0.000	1	0.998	0.000	0.000	–
OFD	3.596	1.272	7.995	1	0.005	36.444	3.014	440.657
Crown lengthening	1.017	1.160	0.768	1	0.381	2.764	0.285	26.837
GV/gingivoplasty	−16.103	8569.170	0.000	1	0.999	0.000	0.000	–
Distal wedge	−16.103	7595.757	0.000	1	0.998	0.000	0.000	–
SFOT	3.020	1.460	4.281	1	0.039	20.500	1.173	358.395
LPT	−16.103	4874.114	0.000	1	0.997	0.000	0.000	–
Constant	−5.100	1.003	25.851	1	<0.001	0.006		

*Note*: Delayed implant placement served as the reference category.

Abbreviations: ADM, acellular dermal matrix; ARP, alveolar ridge preservation; BRG, bone replacement graft; CI, confidence interval; EPG, epithelialized palatal graft; GBR, guided bone regeneration; GTR, guided tissue regeneration; GV, gingivectomy, LPT, laser periodontal therapy; OFD, open flap debridement; SCTG, subepithelial connective tissue graft; SES, sinus elevation surgery; SFOT, surgically facilitated orthodontic therapy.

## 4 DISCUSSION

This investigation was conducted to evaluate the effect of operator‐, procedure‐, patient‐, and site‐related factors on complication occurrence following periodontal and implant surgeries. Increased reporting and analysis of treatment complications to shape clinical decision‐making within periodontics has been encouraged (Chambrone & Zucchelli, 2022; Park et al., 2011; Zucchelli et al., 2023). The most impactful data on complications and adverse outcomes will originate from results of controlled clinical research. Observational investigations, including the current study, are subject to biases and confounding factors that may lead to spurious associations. Nevertheless, this study involved a relatively large number of observations, and a retrospective analysis of complication occurrence was accomplished without risk to any patient. Moreover, findings from the present study can be placed in context of previous studies reporting complication occurrence in other patient samples (Askar et al., 2019; Barone et al., 2006; Fontana et al., 2011; Hernández‐Alfaro et al., 2008; Lim et al., 2018; Moreno Vazquez et al., 2014; Powell et al., 2005; Sakkas et al., 2016; Schwartz‐Arad et al., 2004; Zucchelli et al., 2010).

### 4.1 Postoperative complication occurrence

Although “operator” was ultimately not found to be a statistically significant predictor of postoperative complication occurrence in this study sample, this variable was included in the final regression model due to statitistical significance during hierarchical omnibus testing. Perceived clinical skill has been reported as the primary determinant of patient referrals from general practitioners to periodontists (Kraatz et al., 2017; Park et al., 2011; Zemanovich et al., 2006), and in various dental and medical disciplines, operator skill/experience has been shown to significantly influence treatment outcomes across a wide range of procedure types (Carter, 2003; Cushen & Turkyilmaz, 2013; Kozlovsky et al., 2018; Stulberg et al., 2020). In one study involving general surgeons, operator skill was estimated to account for 25% of the variance in treatment outcomes (Stulberg et al., 2020). In contrast, neither year of training nor clinican significantly influenced postoperative complication occurrence in the present study. All evaluated procedures were completed by second‐ and third‐year periodontics residents. Thus, this study did not involve comparisions across a broad spectrum of operator experience. Additionally, each evaluated produre was directly supervised by a board certified periodontist, and all supervising faculty members had similar training and professional experience. These biases may have mitigated the impact of operator skill/experience in this study.

Procedure type was the only independent variable identified as a statisically significant predictor of postoperative complication occurrence in this study sample. SCTG was associated with threefold increased odds of postoperative complication, and in this study, the most common complication following SCTG was excessive postoperative pain. Pain from the palatal donor site is a known disadvantage of autogenous soft tissue grafting; however, compared with EPG donor sites, SCTG donor sites are typically less uncomfortable (Wessel & Tatakis, 2008). Overthinning the superficial tissue at the donor site can result in necrosis and exposure of underlying connective tissue, permitting topical irritation of the palatal wound (Zucchelli et al., 2010). Periodontists in training may be more likely to overthin the superficial tissue at the palatal harvest site. In addition, palatal graft dimensions have been shown to correlate with postoperative pain (Zucchelli et al., 2010). It is possible that SCTG dimensions influenced the observed postoperative complication occurrence in this study.

GBR and GBR with implant placement were treated as separate procedures for the purpose of this analysis. GBR procedures acomplished in advance of implant surgery may tend to involve larger alveolar ridge deficiencies, whereas small ridge deficiencies may more likely permit simultaneous implant installation. Nevertheless, both GBR alone and GBR with implant placement were associated with increased risk of postoperative complication occurrence in this study. In this sample, the most common complications in GBR procedures with or without implant placement were excessive pain, postoperative infection, and membrane exposure. Multiple authors have associated these complications with GBR (Elgali et al., 2017; Fontana et al., 2011; Lim et al., 2018). Reported soft tissue complication rates following GBR vary substantially—ranging from 0% to 45% (Lim et al., 2018). Membrane type (absorbable vs. nonabsorbable) (Elgali et al., 2017; Urban, 2017) and operator skill (Lim et al., 2018) have been suggested as factors influencing this metric. In particular, chemically cross‐linked collagen membranes and nonabsorbable polytetrafluoroethylene membranes have been associated with high exposure risk (Elgali et al., 2017).

The postoperative complication findings observed in the present study are comparable with results of a previous retrospective investigation involving 3900 procedures (Askar et al., 2019). The reported overall postoperative complication rates were 20.1% and 15.2% in the previous and present studies, respectively (Askar et al., 2019). The surgical procedures evaluated in the two cohorts were similar, although the present analysis included SFOT, ADM, ARP, and LPT rather than third molar surgery. Askar and colleagues (2019) included postoperative chlorhexidine rinse as a variable (Askar et al., 2019). However, in the present study, 0.12% chlorhexidine gluconate was utilized for plaque control following every surgical intervention, until the patient's normal oral hygiene regimen could be reinstated. The present study also categorized compound procedures such as implant placement + GBR, whereas the previous study excluded patients receiving more than one surgical intervention. Additionally, the present study analyzed consecutive surgical procedures accomplished by eight periodontics residents. Thus, the number of procedures assessed in each surgical category varied. In contrast, the previous study involved assessment of 300 records for each procedure of interest, establishing groups of equal size before completing the analysis. In the previous study, the procedures were predominantly performed by supervised residents/students in various dental disciplines, while the procedures in the present study were performed by supervised periodontics residents only. Whereas Askar and colleagues analyzed surgical procedures performed in a university setting, patients in the present study were predominantly active duty military service members, with a small number of military retirees. Despite dissimilar patient populations and substantive methological differences, the two most common postoperative complications in both cohorts were dentin hypersensitivity and excessive pain.

In the present study, smoking did not predict complication occurrence. Smokers with periodontitis are known to respond less favorably to surgical and nonsurgical periodontal therapy (Preber & Bergström, 1986; Tonetti et al., 1995). Cigarette smoking has been associated with reduced implant survival (Anner et al., 2010; Heitz‐Mayfield & Huynh‐Ba, 2009) complications following implant surgery (Schwartz‐Arad et al., 2002), and inferior clinical outcomes in multiple hard and soft tissue grafting procedures (Erley et al., 2006; Horváth et al., 2013; Lindfors et al., 2010; Miller, 1987; Patel et al., 2012; Zitzmann et al., 1999). Increased risk of alveolar osteitis and excessive pain has also been found in cigarette smokers receiving third molar surgery (Schwartz‐Arad et al., 2017). Procedures involving implanted autogenous tissues and biomaterials appear particularly susceptible to the detrimental effects of smoking. For example, the variance in root coverage outcomes for smokers versus nonsmokers appears pronounced in SCTG‐based procedures but may be less significant when the coronally advanced flap technique is employed without implanted autogenous or allogeneic tissue (Chambrone et al., 2009; Chambrone & Tatakis, 2015; Erley et al., 2006). The lack of effect of smoking observed in the present study may reflect clinician bias against procedures involving implanted tissues/biomaterials in smokers, particularly when other risk factors were present. Additionally, in this retrospective study, details of subject smoking experience were not obtainable. The smoking variable included only two levels—smoker and nonsmoker. Defining categories such as light smoker, heavy smoker, and former smoker was not feasible. It is possible that heavy smokers were underrepresented in the present sample.

Diabetic status also was not found to be a statistically significant predictor of complication occurrence in this study. Only 40 diabetic patients were included in the analysis. Generally, these patients appeared well‐controlled (median HbA1c 6.65%), with only three patients exceeding HbA1c of 8%. The lack of effect of diabetes on complication occurrence observed in the present study may reflect relatively low HbA1c levels in most patients and conservative treatment planning for this cohort.

For many procedure types evaluated in this study, perioperative antibiotic use remains controversial. Reported rationale for perioperative antibiotic use includes prevention of postoperative infection (Askar et al., 2019; Lodi et al., 2021), reduction in postoperative pain and swelling (Askar et al., 2019; Lodi et al., 2021), acceleration of healing (Askar et al., 2019), and enhancement in implant survival (Esposito et al., 2013). However, the effect size of antibiotic use in this context is usually small, with some authors finding no benefit, particularly with regard to prevention of postoperative infection (Abu‐Ta'a et al., 2008; Powell et al., 2005; Reddy et al., 2017). Given the observed modest influence on treatment outcomes and the risks associated with antibiotic use, public health experts have advocated curtailing antibiotic use to the extent possible due to concern over development of resistant bacterial strains (American Dental Association Council on Scientific Affairs, 2004; Centers for Disease Control and Prevention CDC, 2013; File et al., 2014). In the present study, no association between perioperative antibiotic use and complication occurrence was identified. The incidence of postoperative infection in the present study (2.2%) is comparable with infection rates reported by Askar and colleagues (1.7%) (Askar et al., 2019) and by Powell and Mealey (2.1%) (Powell et al., 2005). Notably, in the present study, no patients reported a gastrointestial disturbance or other adverse side effects of an antibiotic. In a previous study, 40% of patients receiving clindamycin developed a gastrointestinal disturbance (Askar et al., 2019). Thirty‐one patients in the present sample received clindamycin, with no medication‐related complication. It is possible that this variance is attributable to dosing differences and the age/health of subjects in the two samples. Perioperative antibiotic use is clearly prudent in some circumstances. Clinical decisions regarding this topic should incorporate an individualized risk‐benefit analysis.

### 4.2 Intraoperative complication occurrence

Of the 26 lateral SESs included in this analysis, 10 (38%) involved Schneiderian membrane perforation, consistent with the high incidence of this complication previously reported (Barone et al., 2006; Hernández‐Alfaro et al., 2008; Moreno Vazquez et al., 2014; Sakkas et al., 2016; Schwartz‐Arad et al., 2004). Fortunately, most perforations do not adversely influence implant survival (Barone et al., 2006; Moreno Vazquez et al., 2014; Sakkas et al., 2016; Schwartz‐Arad et al., 2004). However, authors disagree on whether Schneiderian membrane perforation is associated with development of a postoperative complication (Moreno Vazquez et al., 2014; Sakkas et al., 2016; Schwartz‐Arad et al., 2004). Perforation size (Hernández‐Alfaro et al., 2008) and prophylactic antibiotic regimen (Akers et al., 2020) may influence the incidence of postoperative complications following Schneiderian membrane perforation. Of the 51 patients receiving SES in the present study, 43 (84.3%) received perioperative amoxicillin + clavulanate, three (6%) received amoxicillin, and five (10%) received no perioperative antibiotic. In most cases, the antibiotic was started 24 h before the procedure. Although studies identifying the most appropriate prophylactic antibiotic regimen for SES have not been conducted, indirect evidence from the otolaryngology literature suggests that amoxicillin + clavulanate may be more effective than alternative regimens (Akers et al., 2020).

OFD was associated with increased risk of an intraoperative complication in the present study. This procedure is typically planned when periodontal defect severity and configuration are unfavorable for regenerative or resective surgery and when patient‐related factors dictate a simplified surgical approach. In residency, less complex procedures such as OFD may also tend to be performed by less experienced clinicians. In the present study, the observed association between OFD and intraoperative complication occurrence may relate to the low number of observations. Two complications were recorded in 11 OFD procedures. One procedure involved a tear in the mucoperiosteal flap. The other procedure was aborted and rescheduled due to anxiety‐related tachycardia.

## 5 CONCLUSIONS

In this study, SES and OFD were associated with intraoperative complication occurrence, whereas SCTG and GBR were associated with postoperative complication occurrence. Although the operator appeared to influence postoperative complication occurrence, this variable was not found to be statistically significant in the final logistic regression model. Characteristics of the study sample, the study methodology, and biases unique to this investigation may account for smoking, diabetes, operator experience, and perioperative antibiotic use having no detectable effect. The findings of this investigation indicate that procedure type is the predominant factor driving complication occurrence. Evaluation of treatment complications in various contexts represents an essential input into the clinical decision‐making process. As additional treatment complication analyses become available, risk‐informed practitioners will increasingly gain the ability to engage patients in meaningful informed consent processes.

## AUTHOR CONTRIBUTIONS

All authors have contributed substantially to conceptualization of the article, writing the original draft, critical review, and editing. All authors have approved the final version of the manuscript.

## CONFLICT OF INTEREST STATEMENT

The authors declare no conflicts of interest.

## Data Availability

Data sharing not authorized by the Dwight David Eisenhower Army Medical Center Human Protections Office.
